# Successful Treatment of a Symptomatic Vasospasm Following Carotid Artery Stenting With an Intra-arterial Infusion of Fasudil Hydrochloride: A Case Report

**DOI:** 10.7759/cureus.89691

**Published:** 2025-08-09

**Authors:** Hiroshi Saito, Takashi Higa, Masahiko Tanaka, Shizuka Kitajima, Nobuaki Takeda

**Affiliations:** 1 Neurological Surgery, Kawakita General Hospital, Tokyo, JPN

**Keywords:** carotid artery stenting, cerebral vasospasm, fasudil hydrochloride, ischemic complication, local intra-arterial infusion, neurovascular intervention

## Abstract

Symptomatic vasospasm (SVS), a rare complication of carotid artery stenting (CAS), typically improves with medical treatment but can occasionally lead to severe sequelae. We report a case in which SVS following CAS was successfully treated with an intra-arterial infusion of fasudil hydrochloride (FSD). A 74-year-old woman with symptomatic left carotid artery stenosis underwent left CAS using a distal filter protection device. However, she developed a seizure, aphasia, and right hemiparesis seven hours after the procedure.

The following day, magnetic resonance imaging (MRI) of the brain revealed a small high-signal area on diffusion-weighted imaging in the left frontal cortex, along with decreased visualization of the middle cerebral artery (MCA). Angiography demonstrated hypoperfusion due to a vasospasm in the distal segment of the left MCA. Despite ongoing medical treatment, her neurological symptoms worsened. Consequently, we performed a local intra-arterial infusion of FSD, which resulted in a significant improvement of the vasospasm and gradual recovery of neurological function.

On the day following FSD infusion, magnetic resonance angiography revealed an improved visualization of the left MCA. A week after CAS (six days after FSD infusion), the patient’s neurological symptoms had completely resolved, and the abnormal MRI findings had nearly disappeared. She was discharged in the same condition as before undergoing CAS.

These findings suggest that a local intra-arterial infusion of FSD may represent an effective treatment option for SVS that does not respond to medical therapy.

## Introduction

In addition to distal embolism and hyperperfusion syndrome, symptomatic cerebral vasospasm (SVS) has been reported as a rare complication following carotid artery stenting (CAS) [1,2]. SVS, which results in reduced cerebral blood flow, requires prompt diagnosis and treatment, as its perioperative management differs from that of hyperperfusion syndrome and thromboembolism. However, there have been only a few reports on SVS following CAS, and no consensus currently exists regarding its treatment.

We encountered a case of SVS after CAS was performed for symptomatic cervical internal carotid artery stenosis. The patient initially underwent medical therapy; however, her neurological symptoms and radiological findings worsened. An additional local intra-arterial infusion of fasudil hydrochloride (FSD) led to a favorable clinical outcome.

## Case presentation

A 74-year-old woman was brought to the emergency department with dysarthria that had appeared one week prior to admission and repeated falls that had begun two days earlier. She had a medical history of glossodynia, atypical eosinophilia, and hypertension.

On admission, she was alert and oriented, exhibiting mild dysarthria and right hemiplegia. Carotid ultrasonography revealed an isointense plaque at the origin of the right internal carotid artery, with a peak systolic velocity of 403 cm/s. Magnetic resonance imaging (MRI) of the brain demonstrated multiple infarcts in the left parietal lobe, and cervical magnetic resonance angiography (MRA) showed severe stenosis at the origin of the internal carotid artery. Cerebral blood flow, assessed by single-photon emission computed tomography (SPECT), did not show any marked decrease at rest.

The patient was diagnosed with cerebral infarction due to internal carotid artery stenosis. Acute-phase treatment included an intravenous drip infusion of ozagrel sodium and oral administration of antiplatelet agents (aspirin 100 mg/day and clopidogrel 75 mg/day). On day 22 of admission, CAS was performed under general anesthesia. Due to renal dysfunction (serum creatinine: 1.37 mg/dL, estimated glomerular filtration rate: 29.5 mL/min/1.73 m²) observed in blood tests at admission, a 50% diluted contrast medium was used intraoperatively as needed.

**Figure 1:**
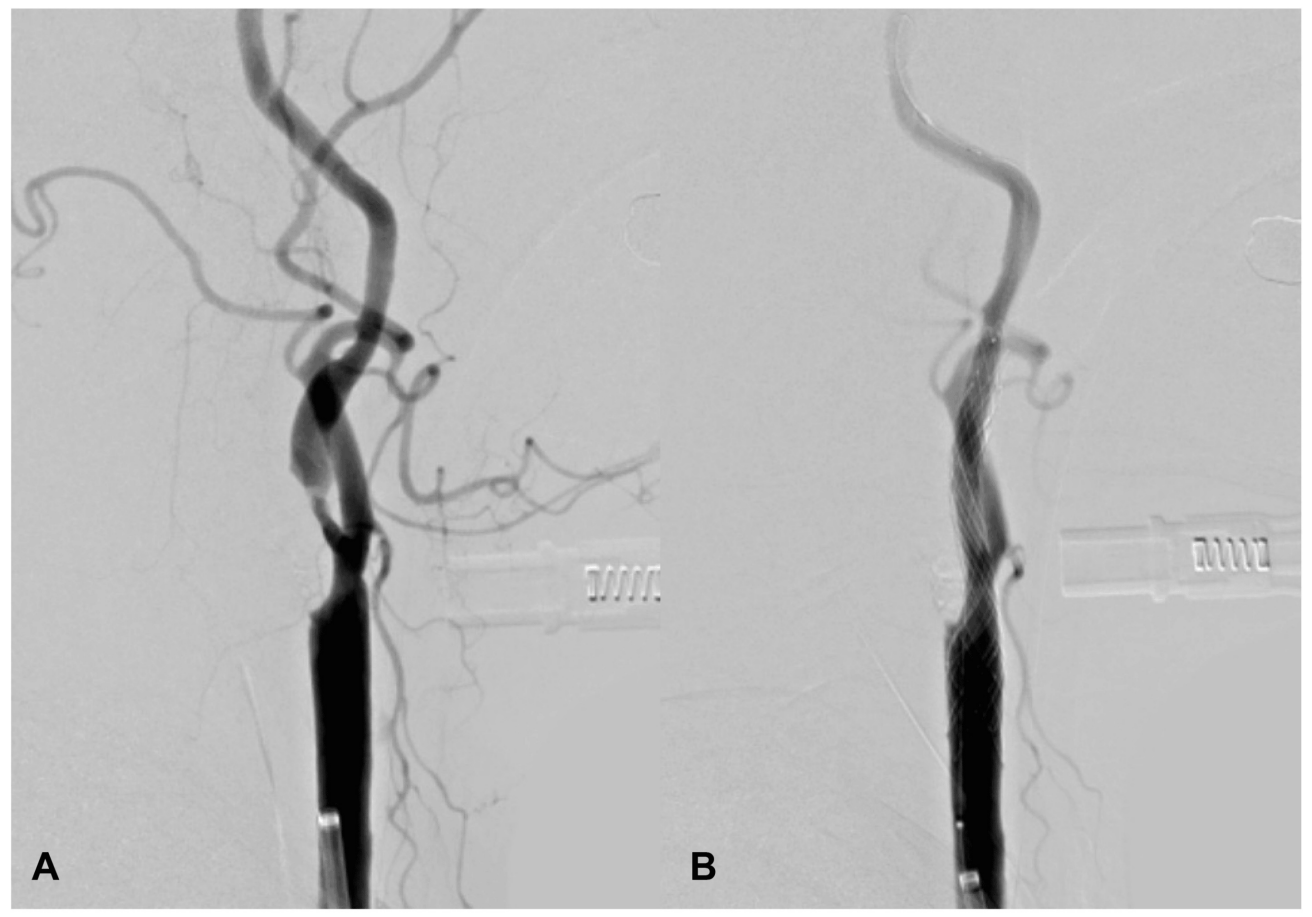
Preoperative and postoperative digital subtraction angiography (A) Preoperative left common carotid angiogram shows severe stenosis in the left internal carotid artery; (B) A 10 mm × 20 mm CASPER stent (Terumo, Tokyo, Japan) was placed to cover the stenotic lesion with a distal filter protection device. Post-procedural angiogram with a twofold diluted concentration contrast medium showed good dilatation of the left internal carotid artery after insertion of the stent.

An 8-Fr OPTIMO EPD balloon guiding catheter (Tokai Medical Products, Aichi, Japan) was placed in the left common carotid artery, and a FilterWire EZ™ (Boston Scientific, Marlborough, MA) was used for distal protection. Pre-dilation was conducted using a 3.0 mm × 40 mm Rx-Genity balloon catheter (Kaneka Medics, Osaka, Japan), a 10 mm × 20 mm CASPER Rx stent (Terumo, Tokyo, Japan) were deployed, and post-dilation was conducted using a 4.5 mm × 20 mm Rx-Genity balloon catheter. Good vessel dilation was achieved (Figure 1).

No bradycardia or hypotension occurred during the procedure, and no debris was captured in the retrieved filter. Immediate postoperative digital subtraction angiography (DSA) showed no abnormalities in the intracranial vessels.

Postoperatively, no new neurological symptoms were observed. The patient was started on continuous argatroban infusion, and blood pressure was managed in the intensive care unit. However, seven hours after the procedure, she developed aphasia, right hemiparesis, and seizures. MRI of the brain did not reveal new infarcts, but the left middle cerebral artery (MCA) was poorly visualized. At that point, the patient did not report any headache. The etiology of her condition remained unclear. Cilostazol and anticonvulsants were initiated in consideration of a possible embolic complication; however, her neurological condition continued to deteriorate the following day.

**Figure 2:**
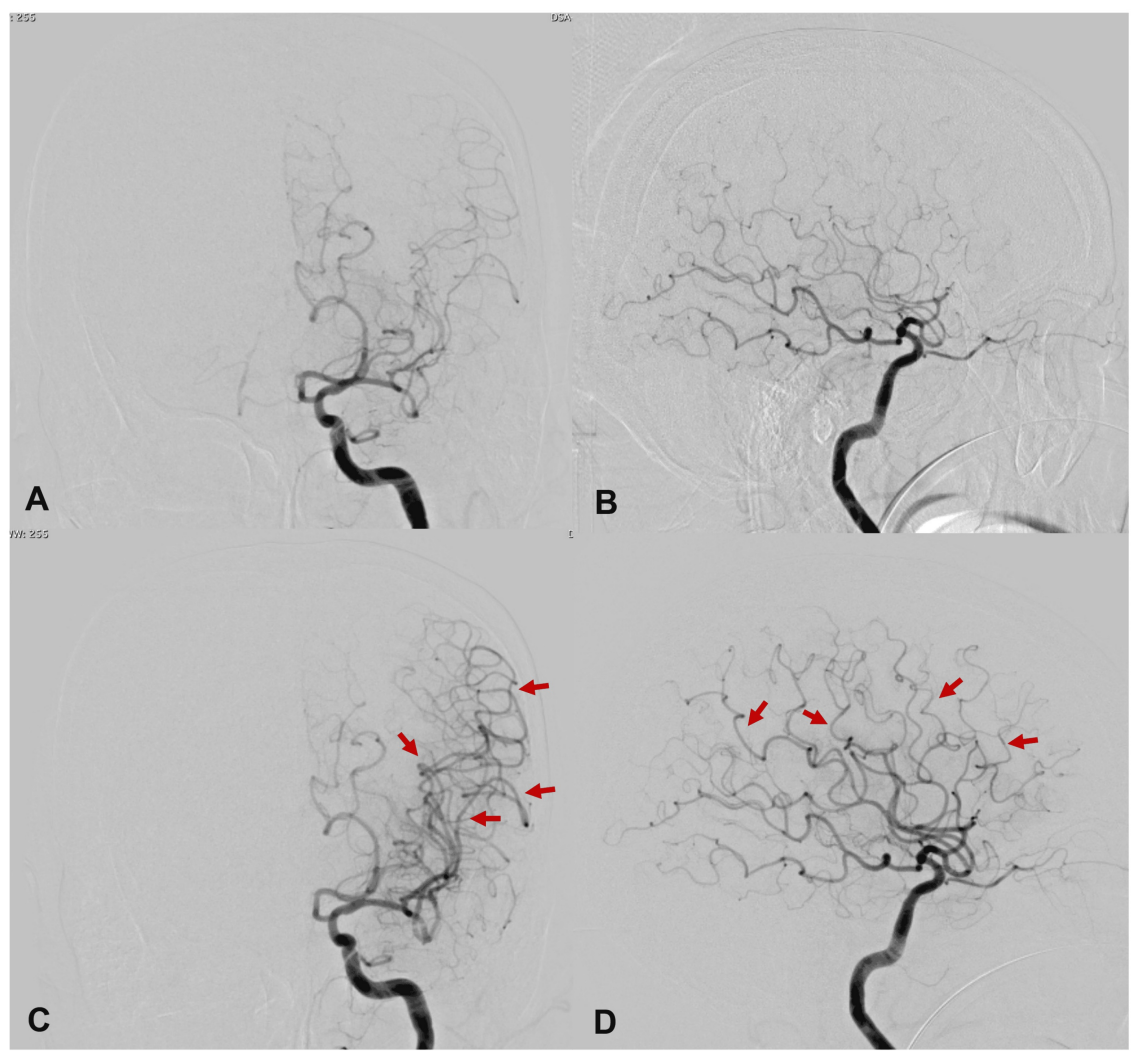
Left internal carotid angiogram performed the day after stenting showing the cerebral vasospasm Internal (A) cerebral anteroposterior view, and (B) lateral view; (C, D) Angiography after local intra-arterial infusion of fasudil hydrochloride shows immediate and marked improvement of the distal middle cerebral artery vasospasm (red arrows).

A repeat MRI of the brain showed further reduction in MCA visualization and a small high-signal area in the left frontal cortex on diffusion-weighted imaging. DSA revealed diffuse narrowing of the distal MCA, although there was no apparent shadow defect in the stent. Based on these findings, the patient was diagnosed with ischemic symptoms secondary to cerebral vasospasm. Due to worsening neurological symptoms and imaging findings, endovascular treatment was initiated. A microcatheter was advanced into the MCA (at the M1 origin) via a diagnostic 5-Fr catheter, and 2 mg of nicardipine (0.1 mg/ml) was administered by an intra-arterial infusion over approximately 12 min. However, no improvement in the vasospasm was observed. Consequently, 30 mg of FSD (1.5 mg/ml) was administered intra-arterially over approximately 12 min, resulting in an immediate improvement in the vasospasm (Figure 2).

The patient, who had been completely aphasic prior to the FSD infusion, began to show improvement in both aphasia and hemiplegia afterward. MRI of the brain performed the day after the local infusion (day two after CAS) also showed improved visualization of the MCA.

**Figure 3:**
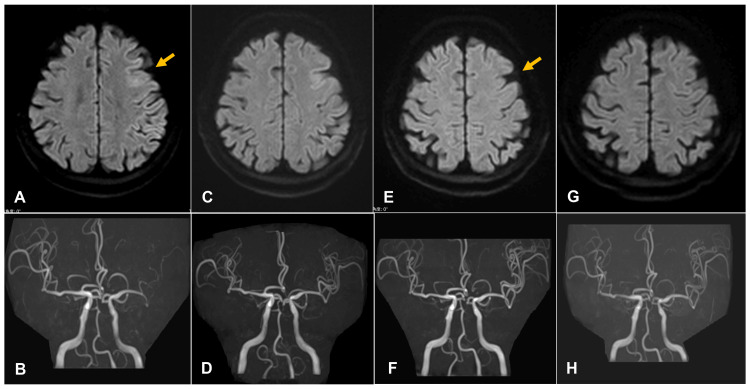
Changes in the image findings CAS, carotid artery stenting; DWI, diffusion-weighted imaging; FSD, fasudil hydrochloride; MCA, middle cerebral artery; MRI, magnetic resonance imaging. (A,B) MRI obtained the day after CAS, when symptoms worsened, shows a small high-signal area on DWI in the left frontal cortex (yellow arrow), with decreased vessel visualization. (C,D) MRI performed the day after local intra-arterial infusion of FSD shows improved visualization of the MCA and resolution of vasospasm. (E,F) MRI taken one week after CAS shows improvement in the abnormal signal (yellow arrow), with slightly increased MCA prominence. (G,H) Follow-up MRI shows normalization of findings.

Simultaneously, hyperdynamic therapy with dobutamine was initiated. SPECT, performed three days after CAS, revealed hyperperfusion, leading to discontinuation of cilostazol and dobutamine. By postoperative day seven, the patient’s neurological symptoms had fully resolved, and the high signal are in the frontal cortex had disappeared on diffusion-weighted MRI of the brain (Figure 3).

By postoperative day 14, MRA of the brain showed no abnormalities. The patient was transferred to a rehabilitation hospital in nearly the same condition as prior to CAS.

The patient and her family provided informed consent for the use of clinical data for this case report.

## Discussion

Among the perioperative complications after CAS, ischemic events warrant the most attention [3], with most being thromboembolic in nature. SVS is reported as a rare complication. However, Kang et al. found that asymptomatic cerebral vasospasm occurred in 35.7% of patients undergoing CAS for severe internal carotid artery stenosis, suggesting that vasospasm may be more common than previously believed when asymptomatic cases are considered [4]. The mechanisms underlying SVS after CAS may include (1) mechanical stimulation caused by the dispersal of microemboli or thrombi from the stenting site [5]; (2) excessive vasoconstriction due to a sudden increase in cerebral blood flow in the setting of impaired cerebral autoregulation, resulting from chronic hypoperfusion [4,6]; and (3) reversible cerebral vasoconstriction syndrome (RCVS) [1,7,8]. SVS typically develops within two days after CAS; however, delayed onset, ranging from two weeks to one month, has also been reported [1,7,8]. Some reports have described SVS occurring in patients with no preoperative reduction in cerebral blood flow on resting SPECT [5,7], as was the case in our patient. Similarly, SVS may occur without the characteristic headache commonly seen in RCVS [2]. The diversity of clinical presentations and imaging findings makes it difficult to explain SVS based on any single mechanism, and its pathogenesis remains unclear.

In our case, the patient developed cerebral ischemic symptoms seven hours after CAS. Although MRI of the brain revealed a small high-signal area in the left frontal cortex on diffusion-weighted imaging, the left MCA was poorly visualized on MRA of the brain, and her neurological symptoms were severe. Differential diagnoses included in-stent thrombosis, seizure-induced Todd’s paresis, and primary central nervous system vasculitis. In-stent thrombosis was ruled out by DSA examination during intra-FSD local intra-arterial infusion therapy. Todd’s paresis could not be completely ruled out since an electroencephalography was not performed, but the change in the MRA findings over time cannot be explained. Primary central nervous system vasculitis is diagnosed by brain biopsy and the exclusion of other precipitating diseases. In the present case, DSA revealed narrowing of the distal MCA, and the findings were reversible, so the diagnosis was SVS. 

Most previously reported SVS cases have been unilateral, although bilateral cases have been described [9], particularly when the contralateral internal carotid artery is occluded. SVS typically occurs in the vascular territory supplied by the treated artery, which was also observed in this case. Treatments for SVS have included anticoagulants, antiplatelet agents, vasodilators, calcium channel blockers, vasopressors, low-molecular-weight dextran, and neuroprotective agents, used alone or in combination. However, no standardized regimen has been established. While SVS often resolves with medical therapy, serious sequelae have also been reported [10].

Because treatment strategies for thromboembolism and SVS differ, distinguishing between them is essential. DSA is considered the gold standard for ruling out in-stent thrombosis and for evaluating the degree of vasospasm. Given that SVS may occur after CAS, performing DSA promptly upon confirmation of reduced MCA visualization on MRA of the brain enables prompt initiation of therapy. 

Local intra-arterial infusion therapy for SVS following CAS has primarily been used in cases unresponsive to medical treatment. Verapamil [1,8] and FSD [11,12] have been employed in such cases outside and within Japan, respectively. Both agents have shown relatively prompt clinical improvement after local infusion, as was seen in our case (Table 1).

**Table 1:** Summary of previous cases treated with local intra-arterial injection therapy for SVS after CAS CAS, carotid artery stenting; SVS, symptomatic vasospasm; VS, vasospasm; US, United States; F, female; M, male; Rt, right; Lt, left; ACA, anterior cerebral artery; MCA, middle cerebral artery; PCA, posterior cerebral artery; FSD, fasudil hydrochloride; IA, intra-arterial injection; NA, not available

Author	Age/Sex	Laterality/Stenosis (%)	Location of VS	Medication used in IA	Angiographical improvement	Neurological improvement	Time to symptom resolution	Recurrence of SVS
Soltanolkotabi et al. [1] (US)	49/F	Lt/70-80	Lt. ACA/MCA/PCA	Verapamil	+	+	NA	-
Aghaebrahim et al. [8] (US)	60/F	Rt/NA	Rt. ACA/MCA	Verapamil	+	+	NA	NA
Sasaki et al. [11](Japan)	73/M	Rt/70	Rt. MCA	FSD	+	+	22 h	-
Yamashina et al. [12](Japan)	79/M	Rt/85	Rt. MCA	FSD	+	+	23 days	-
Our case (Japan)	74/F	Lt/80	Lt. MCA	FSD	+	+	6 days	-

FSD, a Rho kinase inhibitor, exerts its vasodilatory effect by inhibiting intracellular calcium transport and myosin light chain phosphorylation [13]. It is widely used by intravenous administration in Japan for cerebral vasospasm following subarachnoid hemorrhage [14].

Although local intra-arterial infusion of FSD has been associated with hypotension and seizures, the overall incidence of adverse effects remains low [15]. FSD increases cerebral blood flow in a dose-dependent manner. However, because high intravenous doses can induce hypotension, the recommended maximum intravenous dose is 30 mg per administration. According to Sashida et al., intra-arterial administration of FSD achieves local concentrations 20-50 times higher than those achieved intravenously, enabling a stronger vasodilatory effect at the target site [16]. Calcium channel blockers such as verapamil, nicardipine, and nimodipine are also expected to exert a vasodilatory effect but are more likely to cause systemic hypotension compared with FDS, making them less suitable in cases of vasospasm with reduced cerebral blood flow. In this case, we administered nicardipine before FSD administration, but it is unclear whether the amount used was sufficient to achieve a vasodilatory effect. As long as FSD is not administered in large doses, a decrease in blood pressure is rare, and local intra-arterial injection therapy of FSD is considered to be one of the effective treatments for SVS which cannot be improved by medical treatment.

Furthermore, because SVS after CAS may involve distal vessels that are difficult to detect on MRA [4,17], local intra-arterial infusion of FSD offers the advantage of prompt and targeted treatment once the diagnosis is made. In our case, MRA demonstrated improved visualization of the MCA the day after local FSD infusion, and no recurrence of vasospasm was observed after the drug’s elimination half-life had passed.

Two days after the intra-arterial infusion of FSD, SPECT revealed hyperperfusion in the left hemisphere. As a result, the doses of antihypertensive and antiplatelet medications were reduced to minimize the risk of hemorrhagic complications. Hyperperfusion has been reported to occur following the resolution of cerebral vasospasm [2,5,12]. Because the course of SVS following clinical improvement is not fully understood, it is essential to closely monitor the condition using MRI and cerebral perfusion studies and to adjust the treatment strategy as needed.

Finally, as FSD is approved for intravenous use, its application as a local intra-arterial injection therapy represents an off-label use. Therefore, it is essential to provide patients and their families with a thorough explanation and to obtain informed consent prior to treatment.

## Conclusions

In this report, we presented a case of SVS following CAS that was successfully treated with intra-arterial infusion of FSD. Although rare, cerebral vasospasm with worsening neurological symptoms can occur after CAS. Intra-arterial infusion of FSD may serve as an effective therapeutic option in cases that are refractory to standard medical treatment. This case underscores the importance of early recognition and additional treatment of SVS following CAS, particularly in patients who do not respond to standard medical treatment. Further studies are needed to evaluate the efficacy and safety of an intra-arterial infusion of FSD in the treatment of SVS following CAS.
